# Genetic Basis of Tiller Dynamics of Rice Revealed by Genome-Wide Association Studies

**DOI:** 10.3390/plants9121695

**Published:** 2020-12-02

**Authors:** Shuyu Zhao, Su Jang, Yoon Kyung Lee, Dong-Gwan Kim, Zhengxun Jin, Hee-Jong Koh

**Affiliations:** 1 Department of Plant Science, Plant Genomics and Breeding Institute, Research Institute for Agriculture and Life Sciences, Seoul National University, Seoul 08826, Korea; shuyuzhao@126.com (S.Z.); oryzasativa@snu.ac.kr (S.J.); nknglee2403@snu.ac.kr (Y.K.L.); 2 Department of Agronomy, College of Agriculture, Northeast Agricultural University, Harbin 150030, China; zxjin326@hotmail.com; 3 Department of Bioindustry and Bioresource Engineering, Department of Molecular Biology and Plant Engineering Research Institute, Sejong University, Seoul 05006, Korea; kimdg@sejong.ac.kr

**Keywords:** rice tillering, tiller number, productive tiller number, heading date, phase transition, genome-wide association study

## Abstract

A tiller number is the key determinant of rice plant architecture and panicle number and consequently controls grain yield. Thus, it is necessary to optimize the tiller number to achieve the maximum yield in rice. However, comprehensive analyses of the genetic basis of the tiller number, considering the development stage, tiller type, and related traits, are lacking. In this study, we sequence 219 Korean rice accessions and construct a high-quality single nucleotide polymorphism (SNP) dataset. We also evaluate the tiller number at different development stages and heading traits involved in phase transitions. By genome-wide association studies (GWASs), we detected 20 significant association signals for all traits. Five signals were detected in genomic regions near known candidate genes. Most of the candidate genes were involved in the phase transition from vegetative to reproductive growth. In particular, *HD1* was simultaneously associated with the productive tiller ratio and heading date, indicating that the photoperiodic heading gene directly controls the productive tiller ratio. Multiple linear regression models of lead SNPs showed coefficients of determination (*R*^2^) of 0.49, 0.22, and 0.41 for the tiller number at the maximum tillering stage, productive tiller number, and productive tiller ratio, respectively. Furthermore, the model was validated using independent japonica rice collections, implying that the lead SNPs included in the linear regression model were generally applicable to the tiller number prediction. We revealed the genetic basis of the tiller number in rice plants during growth, By GWASs, and formulated a prediction model by linear regression. Our results improve our understanding of tillering in rice plants and provide a basis for breeding high-yield rice varieties with the optimum the tiller number.

## 1. Introduction

Tiller number in rice is the major trait determining plant architecture. An excessive tiller number causes too many unproductive tillers, a reduced leaf area, and a reduction in photosynthetic efficiency by mutual shading. By contrast, too few tillers lead to low biomass production and a deficiency in the grain filling capacity and carbohydrate production [1]. It is important to minimize unproductive tillers and increase the sink size to improve the harvest index in rice, as in the case of China’s super hybrid rice and the IRRI new plant type rice [2]. Tillers are formed from axillary buds, which are derived from the axillary meristems generated on the culm in the leaf axil [3]. Several genes directly regulate axillary meristem initiation, consequently affecting branching in rice. *LAX PANICLE1* (*LAX1*), which encodes a basic helix-loop-helix (bHLH) transcription factor, is the main regulator of axillary meristem formation in rice [4]. *lax1* mutant plants show reduced branching in both vegetative and reproductive growth, with suppressed tillering and panicle branches. *MONOCULM1* (*MOC1*) encodes a transcription factor in the GRAS family and is also involved in axillary bud regulation and rice tillering. *moc1* mutants show completely defective tillering, producing only one main culm [5].

Rice tillering is also influenced by genes involved in phytohormone signaling, such as auxins, cytokinins, strigolactone, and brassinosteroids. The auxin efflux carrier gene *OsPIN1* is involved in polar auxin transport. Transgenic plants with low *OsPIN1* expression show a reduced number of adventitious roots and a significantly increased number of tillers [6]. *Cytokinin oxidase/dehydrogenase 2* (*OsCKX2*) encodes a cytokinin oxidase in rice. The suppression of *OsCKX2* expression by RNAi enhances growth and productivity by increasing the tiller number and grain weight [7]. *DWARF 53* (*D53*) is a substrate of the SCF^D3^ ubiquitination complex and acts as a repressor of strigolactone signaling [8]. In *d53* mutants, strigolactone signaling is blocked, leading to the dwarf and high-tillering phenotype. *Receptor-like cytoplasmic kinases 57* (*OsRLCK57*) is involved in the modulation of brassinosteroid signaling. The transgenic lines silenced for *OsRLCK57* expression by RNA interference results in significant reductions in tillers and panicle secondary branching [9].

In addition, growth phase transitions are a major determinant of rice tillering patterns. Axillary buds produce tillers during the vegetative stage. After the phase transition to the reproductive stage, the shoot apical meristem becomes an inflorescence meristem, and tiller development is delimited [10]. Late heading results in a long duration of vegetative growth, high biomass production, extended tillering, and a low harvest index [11,12]. A short vegetative growth phase (i.e., early heading) results in insufficient biomass production [13]. Rice is a photoperiod-sensitive species with a short-day requirement for heading [14]. Previous studies have revealed several photoperiod-responsive genes. *Heading date 1* (*HD1*), an ortholog of *Arabidopsis CONSTANS* (*CO*), is a photoperiod-responsive flowering gene [15,16]. *HD1* acts as an activator of *HEADING DATE 3a* (*HD3a*) under short-day conditions, resulting in heading [17]. *TIMING OF CAB EXPRESSION 1* (Os*TOC1*), an ortholog of *TOC1* in *Arabidopsis*, is an important circadian clock component and photoperiodic heading regulator [18]. *Hairy meristem 1* and *Hairy meristem* 2 (*OsHAM1* and *OsHAM2*) regulate the vegetative to reproductive phase change [19]. O*sHAM1* and *OsHAM2* are regulated by osa-miR171. The down-regulation of these genes delays heading, thus increasing tiller numbers.

Genome-wide association studies (GWASs), using high-quality single nucleotide polymorphisms (SNPs), can be used to dissect the genetic basis of complex agronomic traits [20]. Loci associated with natural variation in the tiller number have been identified by GWASs using several panels. Huang et al. conducted a GWAS using 671,355 SNPs and 373 accessions classified as the indica landrace, and detected eight association signals for tiller numbers at the full-ripe stage [21]. Another GWAS successfully identified 13 loci associated with the tiller number at the booting stage using 4136 SNPs detected in 469 indica accessions [22]. More recently, a GWAS using 700,000 SNPs revealed 23 loci associated with the tiller number at the later tillering stage of 350 RDP-2 accessions [23]. However, these previous GWASs have focused on tiller numbers at a single stage, without considering alterations during different stages. To comprehensively understand the genetic basis of tiller numbers, it is necessary to consider developmental stages, tiller types, such as productive or unproductive tillers, and relationships between tiller numbers and other traits.

In this study, we sequenced 219 Korean rice accessions and constructed a high-quality SNP dataset. Then, we investigated natural variation in the tiller number at different developmental stages and heading traits. By a GWAS, we identified the complex genetic regulation of tillering and interactions between tiller numbers and phase transitions in rice plants. We also revealed the contribution of each trait-associated loci to the maximum tiller number, productive tiller number, and ratio by a linear regression analysis. Lastly, using independent japonica accessions, we verified the applicability of the detected loci to molecular breeding aimed at optimizing tiller numbers.

## 2. Materials and Methods

### 2.1. Plant Materials and Phenotype Analysis

**Figure 1:**
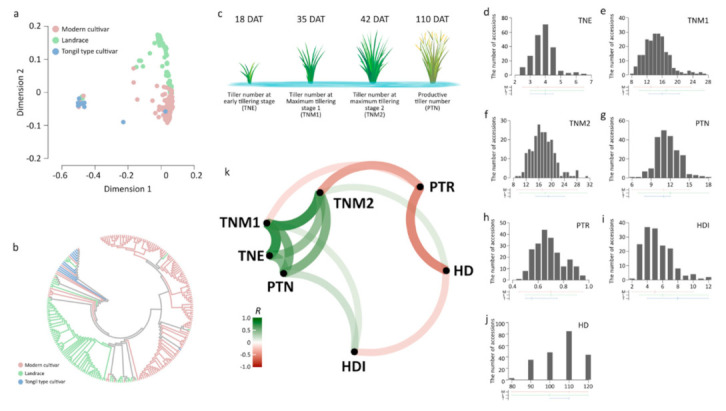
Population structure and phenotypic variation in Korean rice accessions. (**a**,**b**) An multi-dimensional scaling (MDS) plot (**a**) and neighbor-joining (NJ) tree (**b**) were constructed using 37,009 linkage disequilibrium (LD)-pruned single nucleotide polymorphisms (SNPs). Red, modern cultivars; green, landrace; blue, Tongil-type cultivars. (**c**) Tiller number at different developmental stages. Tiller numbers were assessed at 18, 35, 42, and 110 days after transplanting (DAT). (**d**–**j**) Histogram showing distributions of early tillering stage (TNE) (**d**), maximum tillering stage 1 (TNM1) (**e**), maximum tillering stage 2 (TNM2) (**f**), productive tiller number (PTN) (**g**), productive tiller ratio (PTR) (**h**), heading interval (HDI) (**i**), and heading date (HD) (**j**) across accessions. Horizontal and vertical lines below the histogram represent the range and average value, respectively. M, L, and T indicate modern cultivars, landrace, and Tongil-type cultivars, respectively. (**k**) Correlation network for tiller number traits and heading traits. A correlation network was built based on Pearson’s correlation coefficients ^®^ between traits. Each path indicates a correlation between the two traits. The width and transparency of the line denote the strength of the correlation. Weak correlations with *R* between −0.3 and 0.3 are not shown. Green and red represent positive and negative correlations, respectively.

A total of 266 rice accessions, including 219 Korean rice accessions for GWAS and 47 japonica accessions for the verification of linear regression models, were used. Korean rice accessions included 78 landraces, 130 modern cultivars, and 11 Tongil-type cultivars (Table S1). Modern cultivars and Tongil-type cultivars, derived from a cross between temperate japonica and indica, were considered as separate groups. Forty-seven japonica accessions to verify linear regression models were from four origins: Japan, China, Taiwan, and the USA (Table S2). A total of 32 accessions were provided by the National Agrobiodiversity Center (NAC), Rural Development Administration (RDA), South Korea. The other 234 accessions were conserved at the Agricultural Genetic Resource Center, Seoul National University (SNU), Suwon, South Korea. All plant materials were cultivated in an experimental field at SNU, Suwon, South Korea (natural long-day conditions, latitude = 37° N). Thirty-day-old seedlings were transplanted into a paddy field under the following conditions: One plant per hill, 25 plants per row, 15 cm between plants in a row, 30 cm between rows, and three rows per accession. All phenotypes were measured in mid-row plants, excluding plants near other accessions and border plants. Tiller number traits, including tiller numbers at the early tillering stage (TNE), maximum tillering stage 1 (TNM1), maximum tillering stage 2 (TNM2), and productive tiller number (PTN), were measured at 18, 35, 42, and 110 days after transplanting (DAT), respectively (Figure 1c). The productive tiller ratio (PTR) indicated the capacity for developing panicle-emerged tillers from whole tillers. PTR was calculated by the ratio of panicle-emerged tillers (productive tillers) to the maximum potential tillers (PTN/TNM2). The heading date (HD) was defined as the time from the date of sowing to the date at which the first panicle emerged in the plant. Panicle emergence was recoded when a tip of the panicle was visible from the flag leaf sheath. The heading interval (HDI) was defined as the time from the date of emergence of the first panicle to the last panicle in a plant.

### 2.2. NGS Analysis and Genotyping

Total DNA was extracted from 90-day-old leaves of each accession by the CTAB method [24]. DNA was sheared into fragments of 450–500 bp and used for DNA library construction using TruSeq Nano DNA Library Prep kits (Illumina, San Diego, CA, USA) according to the manufacturer’s protocol. The library size distribution was checked using the Agilent Technologies 2100 Bioanalyzer and a DNA 1000 chip (Santa Clara, CA, USA). Prepared libraries were quantified by qPCR according to the Illumina qPCR quantification protocol. Whole genome sequencing data were generated on the Illumina HiSeq X system to generate 2 × 150 bp paired-end reads with a sequencing depth of >10× per sample. Raw reads were processed to remove adaptors and low-quality bases using Trimmomatic v0.38 [25] with the parameters ILLUMINACLIP:2:30:10 SLIDINGWINDOW:4:15 MINLEN:50. Reads were aligned to the rice reference genome (Nipponbare, IRGSP v1.0) [26] using the BWA v0.7.17 MEM algorithm with default parameters [27]. Aligned reads were sorted using samtools v1.9 [28], and duplicates were removed using Picard v2.20.2 [29]. Nucleotide variants were called by the HaplotypeCaller function of GATK v4.1.2 [30] with the parameters—max-missing 0.95—minQ 30—minDP 5. In addition, nucleotide variants with proportions of heterozygous genotypes of >0.05 were filtered using the vc.getHetcount command in GATK v4.12.

### 2.3. Population Structure and Genetic Relationships

Linkage disequilibrium (LD)-based SNP pruning was performed using PLINK v1.9 [31] with the command—indep-pairwise 50 5 0.2. In total, 37,009 LD-pruned SNPs (minor allele frequency [MAF] > 0.05) were obtained for analyses of population structure and genetic relationships. Population structure was revealed by multi-dimensional scaling (MDS) analysis conducted using the MDS function of PLINK v1.9. Neighbor-joining (NJ) trees were constructed to infer genetic relationships using MEGA v7 [32].

### 2.4. Genome-Wide Association Mapping

Only 1,509,362 SNPs with MAF > 0.05 and bi-allelic genotypes were included in the GWASs. All GWASs were performed using linear mixed-models (LMM) implemented in FaST-LMM v2.07 [33]. The genetic similarities were used to estimate random effects. The *p*-value thresholds for genome-wide significance were calculated by dividing the significance level 0.05 by the effective number of independent SNPs (*p*-value of 1.35 × 10^−6^) [34]. LD patterns between lead SNPs and the other SNPs were evaluated using PLINK v1.9 [31] with the -r2 command to calculate pairwise genotype correlations (*r*^2^). Lead SNPs were defined as the SNPs with the lowest *p*-value in loci, including significant SNPs. Haplotypes were constructed using all variants, including SNPs and InDels, without consideration of MAF. Individuals containing at least one missing or/and heterozygous genotype were excluded from the haplotype analysis.

### 2.5. Statistical Analysis

All statistical analyses were performed using R studio v1.2.5033 [35]. Pearson’s correlation coefficients among all phenotypes were calculated without missing observations using the stats package [36]. A correlation network was constructed with *R* > |0.3| using the corrr package [37].

Multiple linear regression models for TNM2, PTN, and PTR were estimated using phenotypes as dependent variables and lead SNPs in the GWAS as independent variables. Independent variables consisted of lead SNPs not only for TNM2, PTN, and PTR, but also for earlier traits with correlations. Since these three terminal traits are affected by traits at the previous growth stage, the directions of relationships between traits were determined based on the sequence of growth stages (Figure 1k). Variables for the best linear equation were selected based on Akaike information criterion (AIC) in a stepwise algorithm implemented in the stats package [36]. The relative importance of independent variables in the linear regression equation was estimated using the lmg method of the relaimpo package [38]. Observed values and predicted values were compared using the predict function of the stats package [36].

### 2.6. Estimation of Heritability

GCTA v1.93 [39] was used to estimate the SNP-based heritability of traits as proportion of phenotypic variance of traits explained by the SNP subset [40]. The genetic relationship matrix to estimate genetic relatedness between individuals was calculated using filtered 1,509,362 SNPs. Variance in phenotypes was calculated by the restricted maximum likelihood method with the genetic relatedness and eigenvectors from a principal component analysis.

## 3. Results

### 3.1. Population Structure of Korean Rice Accessions

An SNP subset obtained by LD-based pruning was used for analyses. An MDS analysis showed that population genetic structures of Korean rice accessions could be classified into two major groups, japonica and Tongil-type accessions, developed from inter-subspecific crosses between indica and temperate japonica (Figure 1a). Japonica accessions were divided into two sub-groups, modern cultivars, and landrace accessions. An NJ tree revealed a similar pattern to that obtained by MDS plot (Figure 1b).

### 3.2. Variation and Changes in the Tiller Number among Growth Stages

The modern japonica cultivar group showed lower TNE and TNM1 than those of japonica landraces and Tongil-type rice (Figure 1d,e). TNM2 was higher in the Tongil-type cultivar group than in the other groups (Figure 1f). PTN and PTR in all Korean rice accessions were mainly in the ranges of 10–11 and 0.65–0.7, respectively. The highest average PTN and PTR values were found in the landrace group (Figure 1g,h). Tongil-type cultivars revealed the lowest phenotypic variation for all traits, but showed the highest average values and variation in HDI (Figure 1i). The average HD was lower in the landrace group than in the other groups (Figure 1j).

Phenotypic relationships among all traits were investigated by correlation analysis (Figure 1k). Three tiller number traits at the vegetative stage (TNE, TNM1, and TNM2) were positively correlated. A positive correlation was found between HD and TNM2. HD was negatively correlated with HDI and PTR. PTR was negatively correlated with TNM2, while the PTN was positively correlated with TNM2 and HDI.

### 3.3. Genome-Wide Association Studies with Factored Spectrally Transformed Linear Mixed Models (FaST-LMM)

**Figure 2:**
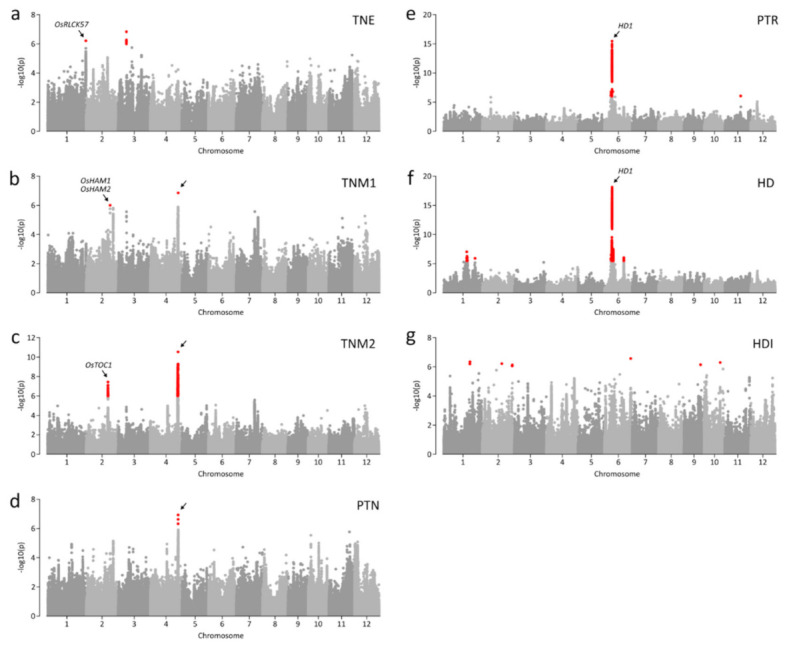
Genome-wide association scan using linear mixed models (LMM). (**a**–**g**) Manhattan plots of genome-wide association studies (GWASs) for TNE (**a**), TNM1 (**b**), TNM2 (**c**), PTN (**d**), PTR (**e**), HD (**f**), and HDI (**g**). Negative log_10_-transformed *p*-values from GWASs using LMM are plotted at positions on each of 12 chromosomes. Red dots indicate significant SNPs (*p* < 1.35 × 10^−6^). Known candidate genes are marked above lead SNPs.

**Figure 3:**
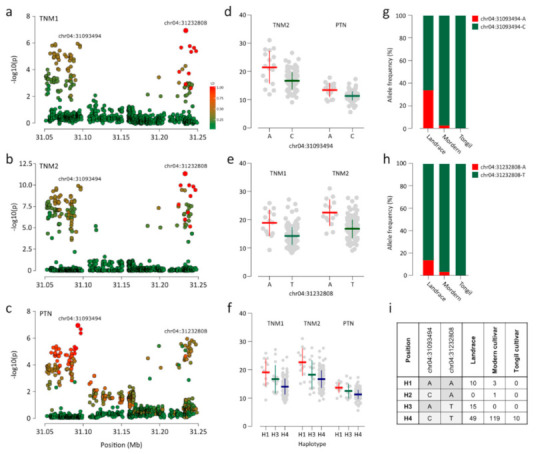
Strong association signal on chromosome 4 for TNM1, TNM2, and PTN. (**a**–**c**) Loci on chromosome 4 near the lead SNP (i.e., the SNP with the highest significance) for TNM1 (**a**), TNM2 (**b**), and PTN (**c**). The color of each SNP indicates the *r*^2^ value for the correlative with the lead SNP. Red and green color intensities indicate stronger and weaker LD (0 to 1). (**d**–**f**) Dot plots for differences in phenotypes by alleles of chr04:31093494 (**d**) and chr04:31232808 (**e**), and the haplotype constructed from two lead SNPs (**e**). (**g**,**h**) Allele frequencies for (**g**) chr04:31093494, (**h**) chr04:31232808, and (**i**) haplotypes constructed from the two lead SNPs.

We detected 20 association signals (*p* ≤ 1.35 × 10^−6^) for all traits (Figure 2; Table 1). Two significant associations were detected for each of TNE, TNM1, and TNM2 (Figure 2a–c). For PTN and PTR, only one and two association signals were detected, respectively (Figure 2d,e). The most significant association (chr01:8365187; *p* = 3.52 × 10^−16^) was detected for HD (Figure 2f). The largest number of loci (i.e., six) were associated with HDI (Figure 2g; Table 1). We found a notable locus at 31.0–31.3 Mb on chromosome 4 where two lead SNPs, chr04:31093494 and chr04:31232808, were associated with TNM2 and PTN and with TNM1 and TNM2 (Figure 3a–c). Although the two lead SNPs were separated by only 150 kb, the LD parameter *r*^2^ was <0.4. Thus, each lead SNP was considered as independent association signals with the traits. Chr04:31093494 and chr04:31232808 were both associated with TNM2 (*p* = 5.82 × 10^−10^ and *p* = 2.88 × 10^−11^, respectively) (Figure 3b). However, only chr04:31232808 was significantly associated with TNM1 (*p* = 1.36 × 10^−7^; Figure 3a), and chr04:31093494 was associated with PTN (*p* = 1.18 × 10^−7^; Figure 3c). The chr04:31232808 A allele was a positive (trait-enhancing) allele, causing higher TNM1 and TNM2 values, and was primarily detected in landrace accessions (Figure 3e,g). Accessions with the chr04:31093494 A allele, mainly landrace accessions, showed higher TNM1 and TNM2 values than those of accessions with the T allele (Figure 3d,h). Four haplotypes were constructed from two alleles for each lead SNP (Figure 3i). Haplotype 1 (H1), consisting of two positive alleles of each lead SNP, showed the highest average TNM1, TNM2, and PTN. Most modern cultivars (96.7%) with H4 showed low TNM1, TNM2, and PTN values.

**Figure 4:**
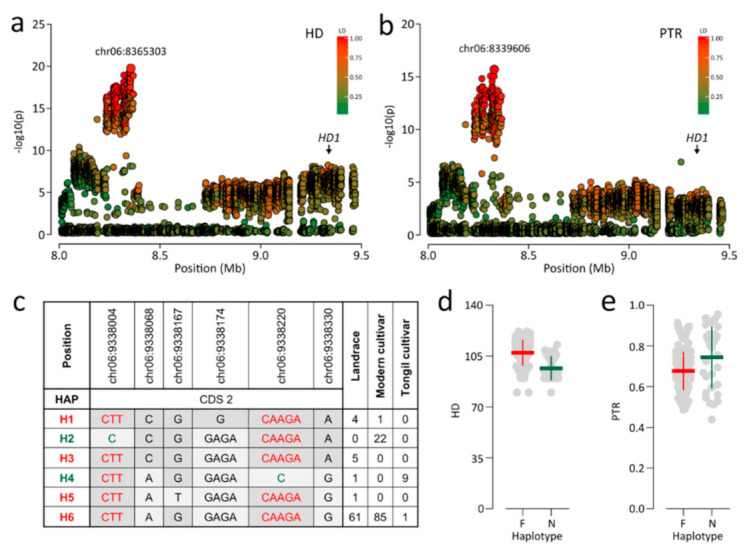
The Loci showing strong association signals for HD and PTR. (**a**,**b**) Genomic regions were showing strong association signals for HD (**a**) and PTR (**b**). The color of each SNP indicates *r*^2^ for the correlation with the lead SNP. Red and green color intensities indicate stronger and weaker LD (0 to 1). (**c**) *HD1* haplotypes. Sequence variants in green are frame-shifting InDels, causing a premature stop codon. Haplotypes in green include nonfunctional alleles led by a premature stop codon. Haplotypes in red denote include functional alleles. (**d**,**e**) Dot plots for differences in HD (**d**) and PTR (**e**) between functional and nonfunctional *HD1*. F, functional alleles; N, Nonfunctional alleles.

Association signals near 8.3 Mb on chromosome 6 exhibited pleiotropic associations with PTR and HD, led by chr06:8339606 (*p* = 3.52 × 10^−16^) and chr06:8365187 (*p* = 1.23 × 10^−20^), respectively (Figure 4a,b). The two lead SNPs were in high LD (*r*^2^ > 0.94) and were separated by about 25 kb. Thus, these lead SNPs were considered the same association signal. *HD1* was located within a region in moderate LD (*r*^2^ > 0.6) with lead SNPs approximately 1 Mb from association signals (Figure 4a,b). *HD1* controls photoperiodic heading [15,16]. Several studies have shown that *HD1* function is affected by whether the *HD1* haplotype includes functional or nonfunctional allele [41,42]. Six *HD1* haplotypes were classified into functional (i.e., H1, H3, H5, and H6) and nonfunctional haplotypes (i.e., H2 and H4) (Figure 4c). In Korean rice accessions, nonfunctional *HD1* was caused by two frame-shifting InDels, chr06:9338004, and chr06:9338220. Functional *HD1* showed a later HD and higher PTR than those for nonfunctional *HD1* (Figure 4d,e).

**Figure 5:**
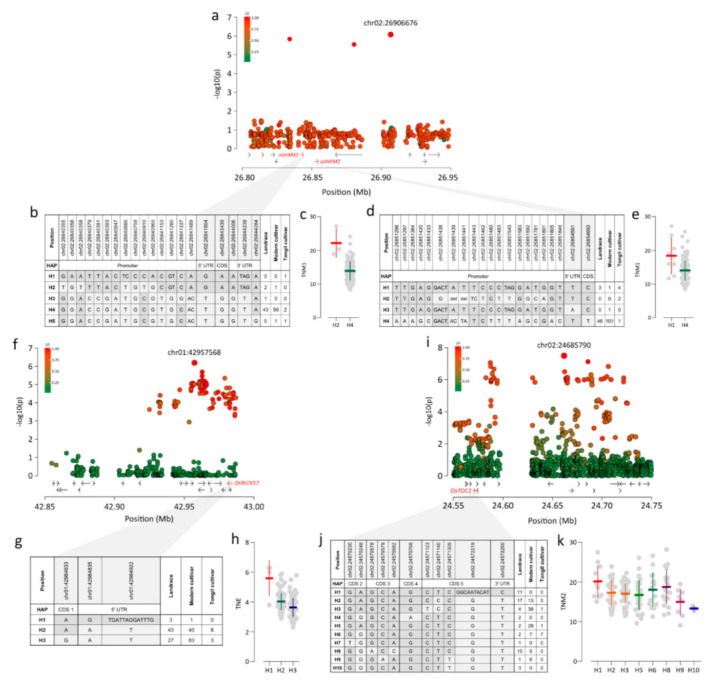
Loci showing strong association signals near candidate genes. Candidate genes located near the lead SNPs (**a**) chr02:26906676, (**f**) chr01:42957568, and (**i**) chr02:24685790. Haplotypes of candidate genes and differences in phenotype among haplotypes. (**b**,**c**) *OsHAM1*. (**d**,**e**) *OsHAM2*. (**g**,**h**) *OsRLCK57*. (**j**,**k**) *OsTOC1*. The color of each SNP indicates the *r*^2^ value for the correlation with the lead SNP. Red and green color intensities indicate stronger and weaker LD (0 to 1). The phenotypic differences were compared for haplotypes detected in more than three accessions.

Chr02:26906676, a lead SNP near 26.9 Mb, was significantly associated with TNM1 (*p* = 9.65 × 10^−7^). We detected two candidate genes, *OsHAM1* and *OsHAM2*, approximately 62 and 52 kb from chr02:26906676 with *r*^2^ > 0.8 (Figure 5a). These genes maintain shoot apical meristem indeterminacy and regulate the vegetative to reproductive phase change **[19]**. Five and four haplotypes were constructed for *OsHAM1* and *OsHAM2*, respectively, using variants in genic regions and the ~1.5 kb promoter region from the 5′ UTR. In Korean accessions, H4 of *OsHAM1* was dominantly detected, accounting for 95% of accessions (Figure 5b). When comparing average TNM1 values among haplotypes, detected in more than two accessions, H2 showed a higher TNM1 than that of H4 (Figure 5c). In *OsHAM2*, H4 was dominantly detected in Korean accessions, accounting for 93% of accessions. H1, only found in eight accessions, conferred the highest average TNM1 (Figure 5d).

We detected association signals for TNE, represented by the lead SNP chr01:42957568 (*p* = 6.11 × 10^−7^), at 42.9 Mb on chromosome 1. A candidate gene, *OsRLCK57*, in strong LD (*r*^2^ > 0.97) was located about 23 kb from chr01:42957568 (Figure 5f). *OsRLCK57* is involved in the modulation of BR signaling and is required to develop tillers and panicle secondary branching [9]. Three *OsRLCK57* haplotypes were constructed by three variants (Figure 5g). H1, found in only four accessions, showed higher TNE values than those of other haplotypes (Figure 5h).

Another locus at 24.5–24.8 Mb on chromosome 2, led by chr02:24685790 (*p* = 7.67 × 10^−8^), was significantly associated with TNM2. Os*TOC1* was located about 113 kb from the lead SNP and was in LD with *r*^2^ > 0.65 (Figure 5i). Os*TOC1* is an important circadian clock component and photoperiodic heading regulator [18]. Ten haplotypes were detected based on variants in the genic region of *OsTOC1.* H1 containing chr02:24572219, an InDel located at the junction between the coding region and 3′UTR, was only detected in landrace accessions, showing the highest TNM2 (Figure 5j,k). H9 and H10 with the chr02:24571309 T allele showed relatively lower TNM2 values than those of the other haplotypes (Figure 5j,k).

### 3.4. Linear Regression Model for Three Tiller-Related Traits

**Figure 6:**
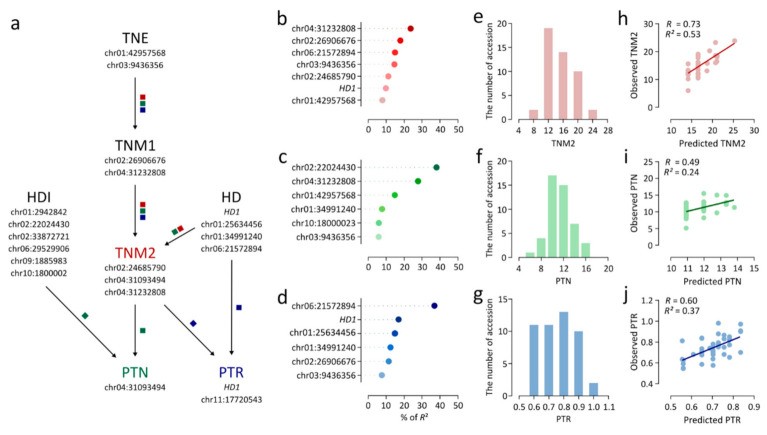
Multiple linear regression models for TNM2, PTN, and PTR. (**a**) Relationships among traits and variants as independent variables for linear regression models. The paths marked by red, green, and blue squares indicate the traits and trait-associated variants involved in TNM2, PTN, and PTR as terminal traits, respectively. (**b**–**d**) Percent of variation explained by independent variables in linear regression models for TNM2 (b), PTN (**c**), and PTR (**d**). (**e**,**f**) Phenotypic variance in 47 independent japonica accessions. (**e**) TNM2. (**f**) PTN. (**g**) PTR. (**h**–**j**) Verification of linear regression models using 47 independent japonica accessions. (**h**) TNM2. (**i**) PTN. (**j**) PTR.

The lead SNPs for TNE, TNM1, TNM2, and HD were included in a linear regression analysis of TNM2 (Figure 6a). A linear regression model consisting of seven independent variables effectively explained variation in TNM2 (*R*^2^ = 0.49) (Table 2). As determined by estimates of relative importance (i.e., the contribution of an individual variable to *R*^2^), chr04:31232808 (23.6%) showed the largest contribution to the linear regression model for TNM2 (Figure 6b). In a linear regression analysis of PTN, six lead SNPs were selected as independent variables, explaining 22.3% of the variance in PTN (Table 2). Chr02:22024430 showed the highest relative importance of 38.1% (Figure 6c). chr04:31232808 showed high relative importance for PTN in addition to TNM2, accounting for 21.5% of *R*^2^ (Figure 6c). The linear regression model for PTR, consisting of six independent variables, exhibited an *R*^2^ of 0.41 (Table 2). Chr06:21572894 and *HD1* showed relatively large contributions to the model, with relative importance values of 36.9% and 16.9% (Figure 6d). Furthermore, an independent test population consisting of 47 japonica accessions collected from several countries was employed to verify the accuracy of the linear regression model (Table S2). TNM2, PTN, and PTR were measured in the test population (Figure 6e–g). Predicted values for TNM2, PTN, and PTR were calculated by applying the regression equations to genotype data for the test population, and were compared with observed values. The correlation coefficients I between predicted and observed values of TNM2, PTN, and PTR were 0.73, 0.49, and 0.6, respectively (Figure 6h–j). Linear regression models for TNM2, PTN, and PTR explained 52.7%, 23.8%, and 36.5% of phenotypic variation, respectively, indicating that the models consisting of lead SNPs consistently explained the three traits with similar accuracy in independent temperate japonica accessions (Figure 6h–j).

## 4. Discussion

To identify the genetic mechanisms underlying tiller number, comprehensive investigations involving consecutive observations and accounting for relationships with other traits affecting tillering are necessary. We investigated relationships between tiller numbers and the heading traits, and performed a GWAS to dissect the genetic basis for tiller numbers.

### 4.1. Phenotypic Relationships

We detected a positive correlation between TNM2 and PTN, indicating that TNM2 could directly affect the potential to produce a panicle. TNM2 was also positively correlated with HD, which reflects the transition to reproductive development. A later HD is related to a longer duration of vegetative growth and the continued development of vegetative organs, causing an increase in TNM2. We detected positive correlations between TNM2 and HD, as well as between TNM2 and PTN, but not between HD and PTN. These results suggest that HD is not a direct determinant of PTN and might have an indirect effect via TNM2.

PTN is an important trait affecting grain yield as it directly determines the panicle number per plant. PTN showed a significant positive correlation with HDI (Figure 1k). A longer HDI represents a longer duration of reproductive growth and panicle development. These results suggest that panicle number, derived from PTN, could be increased by nonsynchronous flowering caused by a longer HDI.

Unproductive tillers are involved in discontinuing nutrient and carbohydrate translocation to the tillers from the mother stems; furthermore, they compete with reproductive tillers for nutrients in addition to light [43,44]. Therefore, a high ratio of productive to unproductive tillers (PTR) is considered a desirable trait for high-yielding varieties [2]. We observed a strong negative correlation between HD and PTR (Figure 1k). As mentioned above, HD was positively correlated with TNM2, but was not significantly correlated with PTN. Additionally, PTN was not correlated with PTR. These results indicate that a higher PTR corresponding with an earlier HD could mainly be explained by reducing TNM2, rather than an increase in PTN.

### 4.2. Association Signals for Tillering and Heading

The tiller number at each growth stage is affected by the tillering capacity at earlier stages [45]. Tillering is controlled by the temporal expression of related genes at various development stages. However, some genetic factors are consistently associated with tillering at several stages [46]. In this study, two lead SNPs, chr01:42957568, and chr03:9436356, were associated with TNE (Figure 2a). chr02:26906676 and chr02:24685790 were associated with TNM1 and TNM2, respectively, exhibiting stage specific-associations (Figure 2b,c). Two lead SNPs, chr04:31093494 and chr04:31232808, were simultaneously associated with TNM2 and PTN and with TNM1 and TNM2, respectively. These results indicate that TNE was controlled by stage-specific QTLs detected only at certain stages. However, tiller numbers after the early stage, TNM1, TNM2, and PTN were affected by combinations of stage-specific and -nonspecific QTLs.

*HD1*, known to control photoperiodic heading, was a candidate gene for PTR and HD based on strong association signals within 8.33–8.37 Mb on chromosome 6, although *HD1* was located approximately 1 Mb away from this region (Figure 2e,f). In the present study, nonfunctional *HD1* resulted from two frame-shifting InDels, chr06:9338004, and chr06:9338220 (Figure 4c). However, only the SNP subset excluding InDels was used for the GWAS, and this association may, therefore, have been missed. Thus, we also performed an additional GWAS for HD and PTR using all variants, including InDels. However, the most significant association was still not detected in the *HD1* region. Instead, chr06:8329287 was the lead SNP (*p* = 2.14 × 10^−15^; Figure S1). A similar pattern of association signals for HD have been reported in a previous GWAS using Japanese rice varieties [47] and a diverse collection [48]. The discrepancy between the strongest peak and *HD1* could be explained by the presence of several linked genes that contribute to heading across the region and/or allelic heterogeneity. Since a GWAS is based on independent comparisons of phenotypic variation for each polymorphic site, statistical significance is reduced when there are several causative alleles [47]. Similar to previous reports, we concluded that the most significant association signal was not detected in the *HD1* region, due to allelic heterogeneity. It is notable that nonfunctional *HD1* leads to a higher PTR, as well as early heading (Figure 2e,f). This result supports that earlier heading date confers a higher PTR, as mentioned above, and suggests that PTR could be improved by the allele of *HD1* related to early heading.

Previous reports have shown that variation in the tiller number is mainly explained by genes directly regulating axillary meristem formation [4,5,49] and genes involved in phytohormone signaling [6,7,8,9]. However, in the present study, we found three candidate genes (*OsHAM1*, *OsHAM2*, and *OsTOC1*) involved in the developmental phase transition near lead SNPs associated with TNM1 and TNM2 (Table 1). In rice, heading results from a developmental switch from the vegetative to reproductive phase, and the repression of heading is involved in the maintenance of vegetative growth, including tiller development. These results imply that natural variation in the tiller number in Korean rice accessions is mainly modulated by genes involved in developmental phase transitions, rather than by genes directly regulating tiller development.

### 4.3. Genetic Determinants of TNM2, PTN, and PTR

To obtain a comprehensive understanding of the genetic basis of TNM2, PTN, and PTR, multiple linear regression models were estimated. The low *R*^2^ value for the linear regression model for PTN (0.22) indicated that a relatively small portion of the variance in PTN could be explained by genetic factors associated with the tiller number and heading traits (Table 2). This result suggests that other genetic variants should be additionally considered to sufficiently explain PTN variation. Based on the relative importance of independent variables in linear regression models, TNM2 was largely contributed by chr04:3123280 (23.6%), the lead SNP for TNM1 and TNM2, followed by chr02:26906676 (17.9%), the lead SNP for TNM1 (Figure 6b). For PTN, chr02:22024430, the lead SNP for HDI, was the major contributor (38.1%; Figure 6c). Chr06:21572894, *HD1*, and chr01:34991240, which were associated with HD, were major contributors to PTR, accounting for 36.9%, 16.9%, and 14.6% of *R*^2^, respectively (Figure 6d). These results indicate that genetic variants associated with earlier stage traits and correlated traits could also influence TNM2, PTN, and PTR, together with lead SNPs for terminate traits (TNM2, PTN, and PTR).

In a previous study of progeny populations derived from various crosses, the heritability of tiller numbers was 37.8–97.7%, depending on the genetic background of the parents [50]. The heritability of tiller numbers in single-segment substitution lines varied from 0% to 39.1% throughout plant growth from 7 to 63 days after transplanting (DAT), and the highest heritability was observed at 42 DAT [51]. In the USDA minicore rice diversity panel, SNP-based heritability of tiller numbers at 60 days after emergence was in the range from 20–25% [52]. These results indicate that the heritability of tiller numbers varies substantially depending on the growth stage, genetic population, and estimation method. In the present study, the heritability of tiller numbers varied from 39.2% (TNI; DAT 18) to 77% (TNM2; 42 DAT), showing substantial variation among growth stages (Figure S2). Furthermore, the heritability of the other tiller number traits PTN and PTR was quite high (i.e., 58.1% and 70.1%, respectively), indicating that a higher proportion of variation of tiller number traits could be explained by genetic factors. Linear regression models estimated using significant SNPs could explain 49%, 22%, and 41% of phenotypic variation in TNM2, PTN, and PTR, respectively (Table 2). Taken together, the small proportion of phenotypic variation explained by multiple linear regression models might be attributable to the use of significant SNPs in the GWAS, while heritability was estimated using all SNPs. Regardless, the multiple linear regression models and genetic variants used as independent variables could be effective molecular tools for the prediction of TNM2, PTN, and PTR in rice breeding programs.

## 5. Conclusions

In this study, we dissected the basis of rice tiller numbers by analyzing relationships with heading traits. We also revealed the genetic basis of tiller alterations at different growth stages, using GWASs. Several candidate genes were detected in loci significantly associated with tiller number. *OsRLCK57* involved in tiller development was associated with the tiller number at the early tillering stage. *OsHAM1*, *OsHAM2*, and *OsTOC1*, which were related to the developmental phase transition, were associated with the tiller number at maximum tillering stages. *HD1* controlling flowering time was associated with the productive tiller ratio. Taken together, these results suggest that genes involved in developmental phase transitions, along with gene modulating tiller development, could also determine the rice tillering pattern at different growth stages. Our results provide insight into the genetic basis of overall tillering dynamics.

## Figures and Tables

**Table 1:** SNPs associated with the tiller number and heading traits.

Trait	Chr	Lead SNP	*p*-Value	Candidate Gene	Gene ID ^a^	Description	Reference
TNE	1	chr01:42957568	6.11 × 10^−7^	*OsRLCK57*	*Os01g0973500*	Receptor-like cytoplasmic kinase	[10]
TNE	3	chr03:9436356	1.47 × 10^−7^				
TNM1	2	chr02:26906676	9.65 × 10^−7^	*OsHAM1*; *OsHAM2*	*Os02g0662700*; *Os02g0663100*	Maintenance of shoot apical meristem indeterminacy; Regulation of vegetative to reproductive phase change	[20]
TNM1	4	chr04:31232808	1.36 × 10^−7^				
TNM2	2	chr02:24685790	7.67 × 10^−8^	*OsTOC1*/*OsPRR1*	*Os02g0618200*	Circadian-associated rice pseudo response regulator; Control of flowering time	[19]
TNM2	4	chr04:31093494	5.82 × 10^−10^				
TNM2	4	chr04:31232808	2.88 × 10^−11^				
PTN	4	chr04:31093494	1.18 × 10^−7^				
PTR	6	chr06:8339606	3.52 × 10^−16^	*HD1*	*Os06g0275000*	Zinc finger protein; Control of photoperiodic flowering time	[16,17]
PTR	11	chr11:17720543	9.10 × 10^−7^				
HD	1	chr01:25634456	1.92 × 10^−8^				
HD	1	chr01:34991240	3.30 × 10^−7^				
HD	6	chr06:8365187	1.23 × 10^−20^	*HD1*	*Os06g0275000*	Zinc finger protein; Control of photoperiodic flowering time	[16,17]
HD	6	chr06:21572894	2.37 × 10^−7^				
HDI	1	chr01:29428424	4.54 × 10^−7^				
HDI	2	chr02:22024430	6.07 × 10^−7^				
HDI	2	chr02:33872721	7.49 × 10^−7^				
HDI	6	chr06:29529906	2.72 × 10^−7^				
HDI	9	chr09:18859830	7.24 × 10^−7^				
HDI	10	chr10:18000023	5.13 × 10^−7^				

^a^ Gene ID for the Rice Annotation Project Database (RAP-DB).

**Table 2:** Summary of linear regression equations for TNM2, PTN, and PTR using lead SNPs.

SNP	Coefficient	SD	*t* ^a^	Allele ^b^	
				0	1
**TNM2**					
chr03:9436356	4.51	1.49	3.034 **	A	C
chr01:42957568	1.04	0.48	2.167 *	A	G
chr02:26906676	4.21	1.02	4.122 ***	A	C
chr04:31232808	4.59	1.03	4.467 ***	T	A
chr02:24685790	2.80	1.17	2.389 *	G	A
*HD1*	−2.37	0.67	−3.521 ***	F	N
chr06:21572894	−1.73	0.55	−3.132 **	T	C
Intercept	16.53	0.34	47.949 ***		
*p*	2.20 × 10^−16^				
*R* ^2^	0.49				
**PTN**					
chr03:9436356	1.83	1.02	1.798	A	C
chr01:42957568	0.64	0.30	2.131 *	A	G
chr04:31232808	2.40	0.67	3.581 ***	T	A
chr01:34991240	1.05	0.52	2.026 *	C	A
chr02:22024430	4.13	1.00	4.149 ***	C	T
chr10:18000023	−1.36	0.64	−2.129 *	C	T
Intercept	10.93	0.21	51.902 ***		
*p*	8.68 × 10^−6^				
*R* ^2^	0.22				
**PTR**					
chr03:9436356	−0.09437	0.05	−1.975	A	C
chr02:26906676	−0.0896	0.03	−2.768 **	A	C
*HD1*	0.078779	0.02	3.446 ***	F	N
chr01:25634456	0.053531	0.02	2.883 **	C	T
chr01:34991240	0.053239	0.03	1.872	C	A
chr06:21572894	0.087943	0.02	4.902 ***	T	C
Intercept	0.649476	0.01	66.599 ***		
*p*	7.68 × 10^−15^				
*R* ^2^	0.41				

^a^ Values of *p* < 0.05, 0.01, and 0.001 are denoted by *, **, and ***, respectively. ^b^ Two different alleles were designated 1 and 0. *R*^2^, Coefficient of determination; TNM2, Tiller number at maximum tillering stage 2; PTN, Productive tiller number; PTR, Productive tiller ratio; F, Functional allele; N, Nonfunctional allele.

## Supplementary Materials

The following are available online at https://www.mdpi.com/2223-7747/9/12/1695/s1, Table S1. Information for 219 Korean rice accessions used in the study. Table S2. Information for 47 japonica rice accessions used for the verification of linear regression models. Figure S1. Association signals for HD on chromosome 6 by a GWAS using all variants, including InDels. Figure S2. SNP-based heritability of seven traits.

## References

[1] Peng S., Cassman K., Virmani S., Sheehy J., Khush G. (1999). Yield potential trends of tropical rice since the release of IR8 and the challenge of increasing rice yield potential. Crop Sci..

[2] Peng S., Khush G.S., Virk P., Tang Q., Zou Y. (2008). Progress in ideotype breeding to increase rice yield potential. Field Crops Res..

[3] Tanaka W., Ohmori Y., Ushijima T., Matsusaka H., Matsushita T., Kumamaru T., Kawano S., Hirano H.-Y. (2015). Axillary Meristem Formation in Rice Requires the *WUSCHEL* Ortholog *TILLERS ABSENT1*. Plant Cell.

[4] Komatsu K., Maekawa M., Ujiie S., Satake Y., Furutani I., Okamoto H., Shimamoto K., Kyozuka J. (2003). LAX and SPA: Major regulators of shoot branching in rice. Proc. Natl. Acad. Sci. USA.

[5] Li X., Qian Q., Fu Z., Wang Y., Xiong G., Zeng D., Wang X., Liu X., Teng S., Hiroshi F. (2003). Control of tillering in rice. Nature.

[6] Xu M., Zhu L., Shou H., Wu P. (2005). A PIN1 Family Gene, OsPIN1, involved in Auxin-dependent Adventitious Root Emergence and Tillering in Rice. Plant Cell Physiol..

[7] Yeh S.-Y., Chen H.-W., Ng C.-Y., Lin C.-Y., Tseng T.-H., Li W.-H., Ku M.S.B. (2015). Down-Regulation of Cytokinin Oxidase 2 Expression Increases Tiller Number and Improves Rice Yield. Rice.

[8] Jiang L., Liu X., Xiong G., Liu H., Chen F., Wang L., Meng X., Liu G., Yu H., Yuan Y. (2013). DWARF 53 acts as a repressor of strigolactone signalling in rice. Nature.

[9] Zhou X., Wang J., Peng C., Zhu X., Yin J., Li W., He M., Wang J., Chern M., Yuan C. (2016). Four receptor-like cytoplasmic kinases regulate development and immunity in rice. Plant Cell Environ..

[10] Wang L., Sun S., Jin J., Fu D., Yang X., Weng X., Xu C., Li X., Xiao J., Zhang Q. (2015). Coordinated regulation of vegetative and reproductive branching in rice. Proc. Natl. Acad. Sci. USA.

[11] Cui K., Peng S., Ying Y., Yu S., Xu C. (2004). Molecular Dissection of the Relationships among Tiller Number, Plant Height and Heading Date in Rice. Plant Prod. Sci..

[12] Vergara B.S., Tanaka A., Lilis R., Puranabhavung S. (1966). Relationship between growth duration and grain yield of rice plants. Soil Sci. Plant Nutr..

[13] Yoshida S. (1981). Fundamentals of Rice Crop Science.

[14] Pautler M., Tanaka W., Hirano H.-Y., Jackson D. (2013). Grass Meristems I: Shoot Apical Meristem Maintenance, Axillary Meristem Determinacy and the Floral Transition. Plant Cell Physiol..

[15] Nemoto Y., Nonoue Y., Yano M., Izawa T. (2016). *Hd1*, A *CONSTANS* ortholog in rice, functions as an *Ehd1* repressor through interaction with monocot-specific CCT-domain protein Ghd7. Plant J..

[16] Yano M., Katayose Y., Ashikari M., Yamanouchi U., Monna L., Fuse T., Baba T., Yamamoto K., Umehara Y., Nagamura Y. (2000). Hd1, a major photoperiod sensitivity quantitative trait locus in rice, is closely related to the Arabidopsis flowering time gene CONSTANS. Plant Cell.

[17] Kojima S., Takahashi Y., Kobayashi Y., Monna L., Sasaki T., Araki T., Yano M. (2002). Hd3a, a rice ortholog of the Arabidopsis FT gene, promotes transition to flowering downstream of Hd1 under short-day conditions. Plant Cell Physiol..

[18] Murakami M., Ashikari M., Miura K., Yamashino T., Mizuno T. (2003). The Evolutionarily Conserved OsPRR Quintet: Rice Pseudo-Response Regulators Implicated in Circadian Rhythm. Plant Cell Physiol..

[19] Fan T., Li X., Yang W., Xia K., Ouyang J., Zhang M. (2015). Rice osa-miR171c Mediates Phase Change from Vegetative to Reproductive Development and Shoot Apical Meristem Maintenance by Repressing Four OsHAM Transcription Factors. PLoS ONE.

[20] Huang X., Zhao Y., Wei X., Li C., Wang A., Zhao Q., Li W., Guo Y., Deng L., Zhu C. (2012). Genome-wide association study of flowering time and grain yield traits in a worldwide collection of rice germplasm. Nat. Genet..

[21] Huang X., Wei X., Sang T., Zhao Q., Feng Q., Zhao Y., Li C., Zhu C., Lu T., Zhang Z. (2010). Genome-wide association studies of 14 agronomic traits in rice landraces. Nat. Genet..

[22] Lu Q., Zhang M., Niu X., Wang S., Xu Q., Feng Y., Wang C., Deng H., Yuan X., Yu H. (2015). Genetic variation and association mapping for 12 agronomic traits in indica rice. BMC Genom..

[23] Jiang S., Wang D., Yan S., Liu S., Liu B., Kang H., Wang G.-L. (2019). Dissection of the Genetic Architecture of Rice Tillering using a Genome-wide Association Study. Rice.

[24] Murray M.G., Thompson W.F. (1980). Rapid isolation of high molecular weight plant DNA. Nucleic Acids Res..

[25] Bolger A.M., Lohse M., Usadel B. (2014). Trimmomatic: A flexible trimmer for Illumina sequence data. Bioinformatics.

[26] Sakai H., Lee S.S., Tanaka T., Numa H., Kim J., Kawahara Y., Wakimoto H., Yang C., Iwamoto M., Abe T. (2013). Rice Annotation Project Database (RAP-DB): An integrative and interactive database for rice genomics. Plant Cell Physiol..

[27] Li H., Durbin R. (2009). Fast and accurate short read alignment with Burrows–Wheeler transform. Bioinformatics.

[28] Li H., Handsaker B., Wysoker A., Fennell T., Ruan J., Homer N., Marth G., Abecasis G., Durbin R. (2009). The Sequence Alignment/Map format and SAMtools. Bioinformatics.

[29] Broad Institute Picard Tools. http://broadinstitute.github.io/picard/.

[30] McKenna A., Hanna M., Banks E., Sivachenko A., Cibulskis K., Kernytsky A., Garimella K., Altshuler D., Gabriel S., Daly M. (2010). The Genome Analysis Toolkit: A MapReduce framework for analyzing next-generation DNA sequencing data. Genome Res..

[31] Purcell S., Neale B., Todd-Brown K., Thomas L., Ferreira M.A.R., Bender D., Maller J., Sklar P., de Bakker P.I.W., Daly M.J. (2007). PLINK: A Tool Set for Whole-Genome Association and Population-Based Linkage Analyses. Am. J. Hum. Genet..

[32] Kumar S., Stecher G., Tamura K. (2016). MEGA7: Molecular Evolutionary Genetics Analysis Version 7.0 for Bigger Datasets. Mol. Biol. Evol..

[33] Lippert C., Listgarten J., Liu Y., Kadie C.M., Davidson R.I., Heckerman D. (2011). FaST linear mixed models for genome-wide association studies. Nat. Methods.

[34] Duggal P., Gillanders E.M., Holmes T.N., Bailey-Wilson J.E. (2008). Establishing an adjusted p-value threshold to control the family-wide type 1 error in genome wide association studies. BMC Genom..

[35] Allaire J. (2012). RStudio: Integrated Development Environment for R.

[36] R Core Team (2013). R: A language and Environment for Statistical Computing. https://stat.ethz.ch/pipermail/r-help/2008-May/161481.html.

[37] Kuhn M., Jackson S., Cimentada J. (2020). Corrr: Correlations in R. https://CRAN.R-project.

[38] Groemping U., Matthias L. (2013). Relaimpo: Relative Importance of Regressors in Linear Models. https://github.com/cengel/r_IPgeocode/blob/master/CRANpkgMaintainers.

[39] Yang J., Lee S.H., Goddard M.E., Visscher P.M. (2011). GCTA: A Tool for Genome-wide Complex Trait Analysis. Am. J. Hum. Genet..

[40] Yang J., Bakshi A., Zhu Z., Hemani G., Vinkhuyzen A.A.E., Lee S.H., Robinson M.R., Perry J.R.B., Nolte I.M., van Vliet-Ostaptchouk J.V. (2015). Genetic variance estimation with imputed variants finds negligible missing heritability for human height and body mass index. Nat. Genet..

[41] Takahashi Y., Teshima K.M., Yokoi S., Innan H., Shimamoto K. (2009). Variations in Hd1 proteins, Hd3a promoters, and Ehd1 expression levels contribute to diversity of flowering time in cultivated rice. Proc. Natl. Acad. Sci. USA.

[42] Zhang J., Zhou X., Yan W., Zhang Z., Lu L., Han Z., Zhao H., Liu H., Song P., Hu Y. (2015). Combinations of the Ghd7, Ghd8 and Hd1 genes largely define the ecogeographical adaptation and yield potential of cultivated rice. New Phytol..

[43] Nuruzzaman M., Yamamoto Y., Nitta Y., Yoshida T., Miyazaki A. (2000). Varietal Differences in Tillering Ability of Fourteen Japonica and Indica Rice Varieties. Soil Sci. Plant Nutr..

[44] Ao H., Peng S., Zou Y., Tang Q., Visperas R.M. (2010). Reduction of unproductive tillers did not increase the grain yield of irrigated rice. Field Crops Res..

[45] Pawar S., Radhakrishnan V., Mohanan K.V. (2016). The Importance of Optimum Tillering in Rice-An overview. South Indian J. Biol. Sci..

[46] Xu Y.B., Shen Z.T. (1991). Diallel analysis of tiller number at different growth stages in rice (*Oryza sativa* L.). Theoret. Appl. Genet..

[47] Yano K., Yamamoto E., Aya K., Takeuchi H., Lo P., Hu L., Yamasaki M., Yoshida S., Kitano H., Hirano K. (2016). Genome-wide association study using whole-genome sequencing rapidly identifies new genes influencing agronomic traits in rice. Nat. Genet..

[48] Zhao K., Tung C.-W., Eizenga G.C., Wright M.H., Ali M.L., Price A.H., Norton G.J., Islam M.R., Reynolds A., Mezey J. (2011). Genome-wide association mapping reveals a rich genetic architecture of complex traits in Oryza sativa. Nat. Commun..

[49] Oikawa T., Kyozuka J. (2009). Two-Step Regulation of LAX PANICLE1 Protein Accumulation in Axillary Meristem Formation in Rice. Plant Cell.

[50] Roy S.C., Shil P. (2020). Assessment of Genetic Heritability in Rice Breeding Lines Based on Morphological Traits and Caryopsis Ultrastructure. Sci. Rep..

[51] Liu G., Zeng R., Zhu H., Zhang Z., Ding X., Zhao F., Li W., Zhang G. (2009). Dynamic expression of nine QTLs for tiller number detected with single segment substitution lines in rice. Theor. Appl. Genet..

[52] Qu M., Zheng G., Hamdani S., Essemine J., Song Q., Wang H., Chu C., Sirault X., Zhu X.-G. (2017). Leaf Photosynthetic Parameters Related to Biomass Accumulation in a Global Rice Diversity Survey. Plant Physiol..

