# Supplementary material for: Genetic Basis of Tiller Dynamics of Rice Revealed by Genome-Wide Association Studies
Source: Plants (Basel). 2020 Dec 2;9(12):1695. doi: 10.3390/plants9121695 (PMC7761586; doi:10.3390/plants9121695)
Supplement: Supplementary file 1 [file plants-09-01695-s001.zip › Figure S1.pptx]

## Slide 1
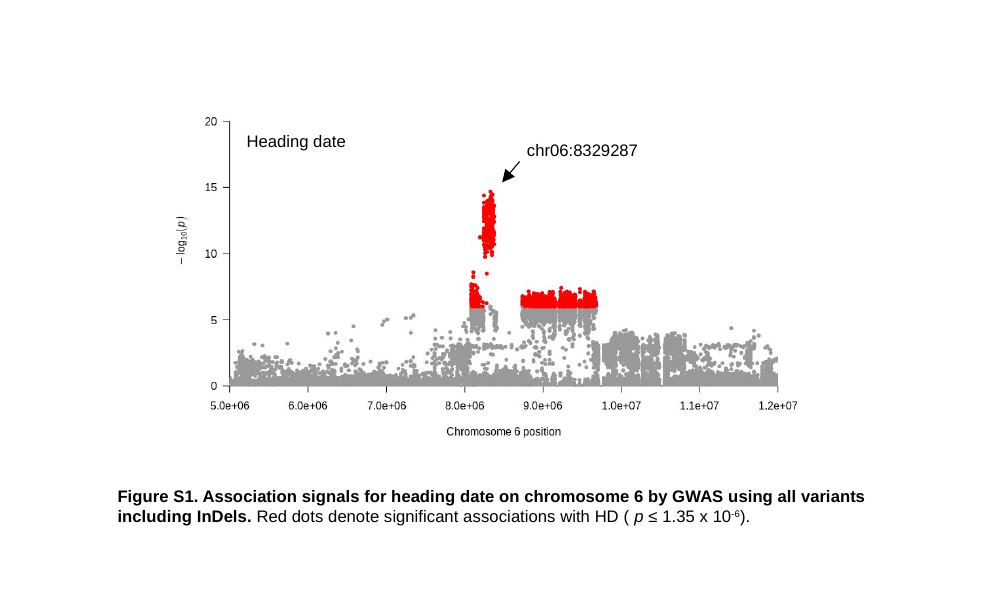

Heading date
chr06:8329287
Figure S1. Association signals for heading date on chromosome 6 by GWAS using all variants including InDels. Red dots denote significant associations with HD ( p ≤ 1.35 x 10^-6^).
