# Supplementary material for: Genetic Basis of Tiller Dynamics of Rice Revealed by Genome-Wide Association Studies
Source: Plants (Basel). 2020 Dec 2;9(12):1695. doi: 10.3390/plants9121695 (PMC7761586; doi:10.3390/plants9121695)
Supplement: Supplementary file 1 [file plants-09-01695-s001.zip › Figure S2.pptx]

## Slide 1
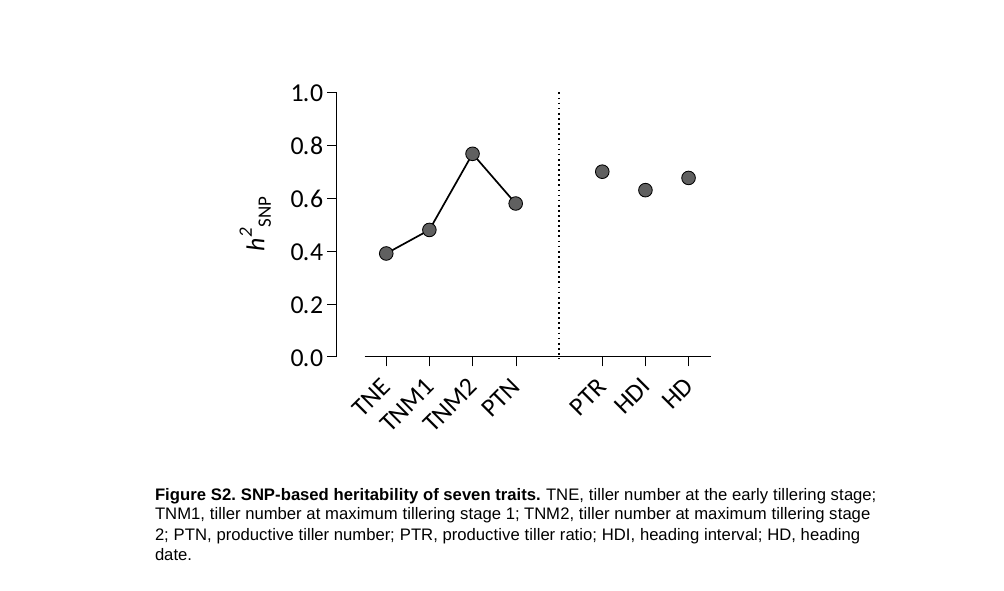

Figure S2. SNP-based heritability of seven traits. TNE, tiller number at the early tillering stage; TNM1, tiller number at maximum tillering stage 1; TNM2, tiller number at maximum tillering stage 2; PTN, productive tiller number; PTR, productive tiller ratio; HDI, heading interval; HD, heading date.
